# From integrative disease modeling to predictive, preventive, personalized and participatory (P4) medicine

**DOI:** 10.1186/1878-5085-5-S1-A49

**Published:** 2014-02-11

**Authors:** Erfan Younesi, Martin Hofmann-Apitius

**Affiliations:** 1 Department of Bioinformatics, Fraunhofer Institute for Algorithms and Scientific Computing (SCAI), Schloss Birlinghoven, 53754 Sankt Augustin, Germany; 2 Rheinische Friedrich-Wilhelms-Universität Bonn, Bonn-Aachen International Center for IT, Dahlmannstr. 2, 53113 Bonn, Germany

## 

With the significant advancement of highthroughput technologies and diagnostic techniques throughout the past decades, molecular underpinnings of many disorders have been identified. However, translation of patient-specific molecular mechanisms into tailored clinical applications remains a challenging task, which requires integration of multi-dimensional molecular and clinical data into patient-centric models. This task becomes even more challenging when dealing with complex diseases such as neurodegenerative disorders. Integrative disease modeling is an emerging knowledge-based paradigm in translational research that exploits the power of computational methods to collect, store, integrate, model and interpret accumulated disease information across different biological scales from molecules to phenotypes. We argue that integrative disease modeling will be an indispensable part of any P4 medicine research and development in the near future and that it supports the shift from descriptive to causal mechanistic diagnosis and treatment of complex diseases. For each ‘P’ in P4 medicine, we demonstrate how integrative disease modeling can contribute to addressing the real-world issues in development of new predictive, preventive, personalized and participatory measures. With the increasing recognition that application of integrative systems modeling is key to all activities in P4 medicine, we envision that translational bioinformatics in general and integrative modeling in particular will continue to open up new avenues of scientific research for current challenges in P4 medicine as outlined in the concluding part of this paper.

